# Comment to: Vegetable peptones as a fetal bovine serum substitute in human deciduous tooth pulp stem cell culture

**DOI:** 10.31744/einstein_journal/2026CE2068

**Published:** 2025-12-05

**Authors:** Naresh Waran Gnanasegaran

**Affiliations:** 1 Cuprina Pte Ltd Singapore Cuprina Pte Ltd, Singapore.

Dear Editor,

The innovative use of vegetable peptones as substitutes for fetal bovine serum (FBS) in stem cell culture presents promising advantages. Replacing FBS addresses major ethical and biosafety concerns, reduces batch variability, and lowers the risk of xenogeneic contamination – factors crucial for translational and clinical cell therapies. Notably, wheat peptone supported equal or superior cell proliferation and osteogenic differentiation than that of FBS, likely because of its high glutamic acid and proline content.

Drawing on our extensive research in stem cell biology and regenerative dentistry, the use of vegetable peptones as FBS substitutes in dental pulp stem cell (DPSC) cultures directly addresses the longstanding limitations of serum-based media. Recurrent challenges with FBS include batch-to-batch variability, undefined components affecting differentiation pathways, immunogenicity, and translational barriers to clinical-grade and patient-specific therapies.^( 1 , 2 )^

Replacing FBS with wheat or soy peptones offers notable advantages.

- Ethical and Regulatory Alignment: Eliminates xenogenic risks and aligns with advanced cell therapy guidelines.
- Reproducibility and Standardization: Defined amino acid profiles – specifically in wheat peptone – enhance consistency across culture batches.
- Enhanced Growth and Differentiation: Wheat peptone supports robust proliferation and osteogenesis, consistent with the regenerative aims documented in our study.
- However, crucial gaps remain, including long-term functional stability, applicability across diverse stem cell types, and elucidation of molecular mechanisms, highlighting the need for further translational research.
- Long-term and Functional Stability: Further studies should address whether these peptone-driven cultures preserve stemness and functional properties (neurotrophic and immunomodulatory effects) – that are crucial for DPSC transplantation and neuronal differentiation models.
- Applicability Across Lineages: Our experience demonstrates that different progenitor cells (mesenchymal and neuronal) may require specific nutritional cues – therefore, cross-validation beyond dental pulp stem cells is essential.
- Molecular Mechanisms: Understanding how specific peptone compositions modulate signaling pathways or the secretome, as explored in earlier publications, may strengthen the mechanistic justification for clinical translation.

In light of this evidence, integrating vegetable-derived peptones, specifically wheat peptone, offers a biologically effective, ethically compliant, and reproducible alternative to FBS for stem cell culture, aligning with clinical translation imperatives.^( 3 )^ Based on our extensive research on optimizing dental pulp stem cell expansion and differentiation, this approach holds significant promise for advancing regenerative therapies.^( 1 , 4 )^ However, rigorous assessment of long-term phenotypic stability, lineage versatility, and mechanistic pathways is essential to ensure safety, efficacy, and regulatory acceptance for future clinical applications.

## Data Availability

The underlying content is contained within the manuscript.
